# Molecular Characterization of *Staphylococcus aureus* Isolates Transmitted between Patients with Buruli Ulcer

**DOI:** 10.1371/journal.pntd.0004049

**Published:** 2015-09-11

**Authors:** Nana Ama Amissah, Monika A. Chlebowicz, Anthony Ablordey, Artur J. Sabat, Caitlin S. Tetteh, Isaac Prah, Tjip S. van der Werf, Alex W. Friedrich, Jan Maarten van Dijl, John W. Rossen, Ymkje Stienstra

**Affiliations:** 1 Department of Internal Medicine/Infectious Diseases, University of Groningen, University Medical Center Groningen, Groningen, the Netherlands; 2 Department of Bacteriology, Noguchi Memorial Institute for Medical Research, University of Ghana, Legon, Ghana; 3 Department of Medical Microbiology, University of Groningen, University Medical Center Groningen, Groningen, the Netherlands; University of Tennessee, UNITED STATES

## Abstract

**Background:**

Buruli ulcer (BU) is a skin infection caused by *Mycobacterium ulcerans*. The wounds of most BU patients are colonized with different microorganisms, including *Staphylococcus aureus*.

**Methodology:**

This study investigated possible patient-to-patient transmission events of *S*. *aureus* during wound care in a health care center. *S*. *aureus* isolates from different BU patients with overlapping visits to the clinic were whole-genome sequenced and analyzed by a gene-by-gene approach using SeqSphere^+^ software. In addition, sequence data were screened for the presence of genes that conferred antibiotic resistance.

**Principal Findings:**

SeqSphere^+^ analysis of whole-genome sequence data confirmed transmission of methicillin resistant *S*. *aureus* (MRSA) and methicillin susceptible *S*. *aureus* among patients that took place during wound care. Interestingly, our sequence data show that the investigated MRSA isolates carry a novel allele of the *fexB* gene conferring chloramphenicol resistance, which had thus far not been observed in *S*. *aureus*.

## Author Summary

Buruli ulcer (BU) is a skin infection caused by *Mycobacterium ulcerans*. The wounds of most BU patients are colonized with different microorganisms, including *Staphylococcus aureus*. This study investigated patient-to-patient transmission events during wound care in a health care center. *S*. *aureus* isolates from patients who visited the health center at the same time points were analyzed using whole-genome sequencing. Analysis of sequence data confirmed transmission of methicillin resistant *S*. *aureus* and methicillin susceptible *S*. *aureus* among patients that took place during wound care.

## Introduction

Buruli ulcer (BU) is a neglected necrotizing skin disease caused by *Mycobacterium ulcerans*, with the highest burden of the disease in West Africa, particularly in Benin, Cote d’Ivoire and Ghana [1]. The disease usually starts as a nodule, plaque, oedema or papule and progresses to form large ulcers with undermined edges if left untreated. It was previously shown that wounds of most BU patients are heavily colonized by many microorganisms, including *Staphylococcus aureus* [2,3].

*S*. *aureus* can be part of the human microbiota colonizing the skin and mucosal membranes without any clinical manifestations. However, once it crosses the skin barrier, or when the host immune system is compromised, this bacterium is able to cause a wide range of diseases, such as skin and soft tissue infections, osteomyelitis, pneumonia, meningitis, or bacteremia [4,5]. Therefore, *S*. *aureus* is considered a dangerous pathogen in both community-acquired and nosocomial infections. Colonization of healthy individuals with multi-drug resistant *S*. *aureus* is regarded as a risk factor for future development of *S*. *aureus* infections that are difficult to treat [6]. The *S*. *aureus* colonization of patients with a serious breach of skin barrier, such as patients with BU, burn wounds or the group of hereditary mechanobullous diseases epidermolysis bullosa (EB), was previously shown to be very high [2,3,7–9]. Molecular typing of *S*. *aureus* isolated from the wounds of BU and EB patients has shown that their wounds often harbor multiple genotypes of this pathogen [3,10].

Recently, several *S*. *aureus* clones have been reported in health care institutions in Ghana with the sequence types (ST) 15, 121 and 152 being the most prevalent as determined by multi-locus sequence typing (MLST) [11]. Notably, health care-associated infections (HAIs) caused by *S*. *aureus* impose a significant burden on patient care as a result of prolonged hospital stays, increased cost of treatments and high morbidity and mortality rates. Current practices implemented to reduce HAIs include cleaning of the hospital environment, hand hygiene and screening and decolonization of patients and health care workers [12–15]. Epidemiological data and molecular typing methods, such as pulsed-field gel electrophoresis (PFGE), MLST, *spa*-typing, multiple-locus variable number tandem repeat fingerprinting (MLVF), and whole-genome sequencing (WGS) of the infecting strains can be used to trace transmission events [16–19]. Each of these typing methods has particular advantages [20]. For example, MLVF is fast, cheap and highly discriminatory [21], while WGS provides additional information on the genetic makeup of investigated isolates on top of a highly discriminatory typing result.

BU patients may be at risk of hospital-associated colonization with *S*. *aureus* due to their frequent visits to particular health care centers for wound care. This represents an additional health risk for these patients, even if they are already colonized with community-acquired *S*. *aureus*. Therefore, the present study was aimed at uncovering possible *S*. *aureus* transmission events among BU patients using MLVF and WGS. Furthermore, WGS was applied to identify antimicrobial resistance (AMR) genes and to screen for mutations in genes that confer certain resistance phenotypes. The results obtained underpin the potential of the combined use of MLVF and WGS for the surveillance of *S*. *aureus* outbreaks in hospital settings.

## Materials and Methods

### Ethics statement

The ethical committee of the Noguchi Memorial Institute for Medical Research (NMIMR) (FEDERAL WIDE ASSURANCE FWA 00001824) approved the use of clinical samples for this investigation. Samples were collected upon written informed consent from adult subjects and a parent or guardian of any child participant on their behalf.

### Bacterial isolates and genomic DNA extraction

A subset of the *S*. *aureus* isolates from BU patients that were previously collected and grouped by MLVF into thirteen clusters (A-M) [3] were selected for WGS. For the present study, isolates were selected from each of the thirteen MLVF clusters including two clusters suspected of patient-to-patient transmission events during wound care (clusters H and F). Screening of BU patients for the presence of *S*. *aureus* had been repeated every two weeks for a period of seven months, which defined the sampling time points t1 to t13 in this study (Table 1). Patients involved in this screening were at different stages of the disease and treatment for BU. All presently investigated *S*. *aureus* isolates were obtained from positive anterior nares and wound cultures of eleven BU patients who attended the Pakro Health Center in the Eastern region of Ghana for antimicrobial therapy (Table 1).

**Table 1:** Frequency of *S*. *aureus* with different *spa*-types isolated during patient visits for wound care.

			12-12-2012	9-1-2013	23-1-2013	6-2-2013	20-2-2013	6-3-2013	20-3-2013	3-4-2013	17-4-2013	9-5-2013	23-5-2013	20-6-2013	4-7-2013
	Patient No.	Start date of treatment	t1	t2	t3	t4	t5	t6	t7	t8	t9	t10	t11	t12	t13
	2	1-12-2012	p	***t786***	**t786**	x	p	x	x	***t786***	**t786**	x	x	x	x
ST88	7	26-12-2012		p	p	t355	***t786***	t084, t939	x	t084, t939, t1096	x	x	t002, t084	x	x
	19	5-1-2013		p	***t786***	x	x	x	x	x	x	x	x	x	x
ST152	7	26-12-2012		p	p	**t355**	t786	t084, t939	x	t084, t939, t1096	x	x	t002, t084	x	x
	5	22-12-2012		p	p	p	p	x	x	x	x	x	**t355**	x	x
	6	12-12-2012	t7835	x	x	x	x	x	x	x	x	x	**t355**	x	x
	3	6-12-2012	p	t084	p	p	x	x	t084	**t084, *t355***	t084, t314	t084	t084	p	p
	10	12-12-2012	p	t355	t355	***t355***	t355	x	x	***t355***	x	x	x	x	x
	11	19-12-2012		***t355***	x	x	t355	x	x	x	x	x	x	x	x
	18	7-1-2013		p	p	p	**t355**	p	p	p	x	x	x	x	x
	24	14-2-2013					**t355**	x	x	x	x	x	x	x	x

‘p’ indicates that a patient visited the health center, but no *S*. *aureus* was detected.

‘x’ indicates that a patient did not visit the health center at the respective time point of sampling.

‘t1’ to ‘t13’ refers to the time points at which samples were collected.

Cells with bold formatting indicate involvement of the respective *S*. *aureus* isolates in transmission events. *spa*-types with italic formatting are isolates that were sequenced.

Genomic DNA was extracted from *S*. *aureus* isolates grown overnight on blood agar by using the Ultraclean microbial DNA isolation kit (mo bio laboratories, Inc, Carlsbad, California, USA) according to the manufacturers’ instructions.

### Whole-genome sequencing, sequence assembly and data analyses

DNA libraries were prepared using the Nextera XT v2 kit (Illumina, San Diego, CA, USA) according to the manufacturers’ instructions and then run on a Miseq (Illumina) for generating paired-end 250-bp reads. *De novo* sequence assembly was performed using CLC Genomics Workbench v7.0.4 (CLC bio A/S, Aarhus, Denmark) after quality trimming (Qs > 28) with optimal word sizes based on the maximum N50 value. The assembled files were imported as Fasta files into SeqSphere^+^ software version 1.1 (Ridom GmbH). The sequence reads were submitted to the National Center for Biotechnology Information GenBank and are available under the BioProject PRJNA283747 and accession numbers: LGAE00000000, LFTW00000000, LFTV00000000, LFTU00000000, LFTT00000000, LFOH00000000, LFOG00000000, LFNS00000000, LFNR00000000, LFNQ00000000, LFNP00000000, LFNO00000000, LFNN00000000, LFNM00000000, LFNL00000000, LFNK00000000, LFNJ00000000, LFNI00000000, LFNH00000000, LFMH00000000, LFMG00000000. The sequence data of the 21 isolates were characterized by using the core genome multilocus sequence typing (cgMLST) consisting of 1,861 genes and 706 *S*. *aureus* accessory genes. The complete sequence of each isolate was analyzed based on gene-by-gene comparison with the reference *S*. *aureus* strain COL (GenBank accession no. NC_002951) and *S*. *aureus* cgMLST target definer function with the default parameters of the software as previously described [22]. Each allele was assigned a number and an allelic typing profile based on the combination of all alleles for each isolate by the software. A dendrogram of the sequenced isolates and two additional reference genomes that represent different sequence types (ST5 [N315 GenBank accession no. BA000018.3] and ST8 [COL]) was generated using an unweighted-pair group method using average linkages (UPGMA). The concordance between the two typing methods was calculated with the Ridom EpiCompare software version 1.0 as described previously [19].

### Transmission events

**Fig 1:**
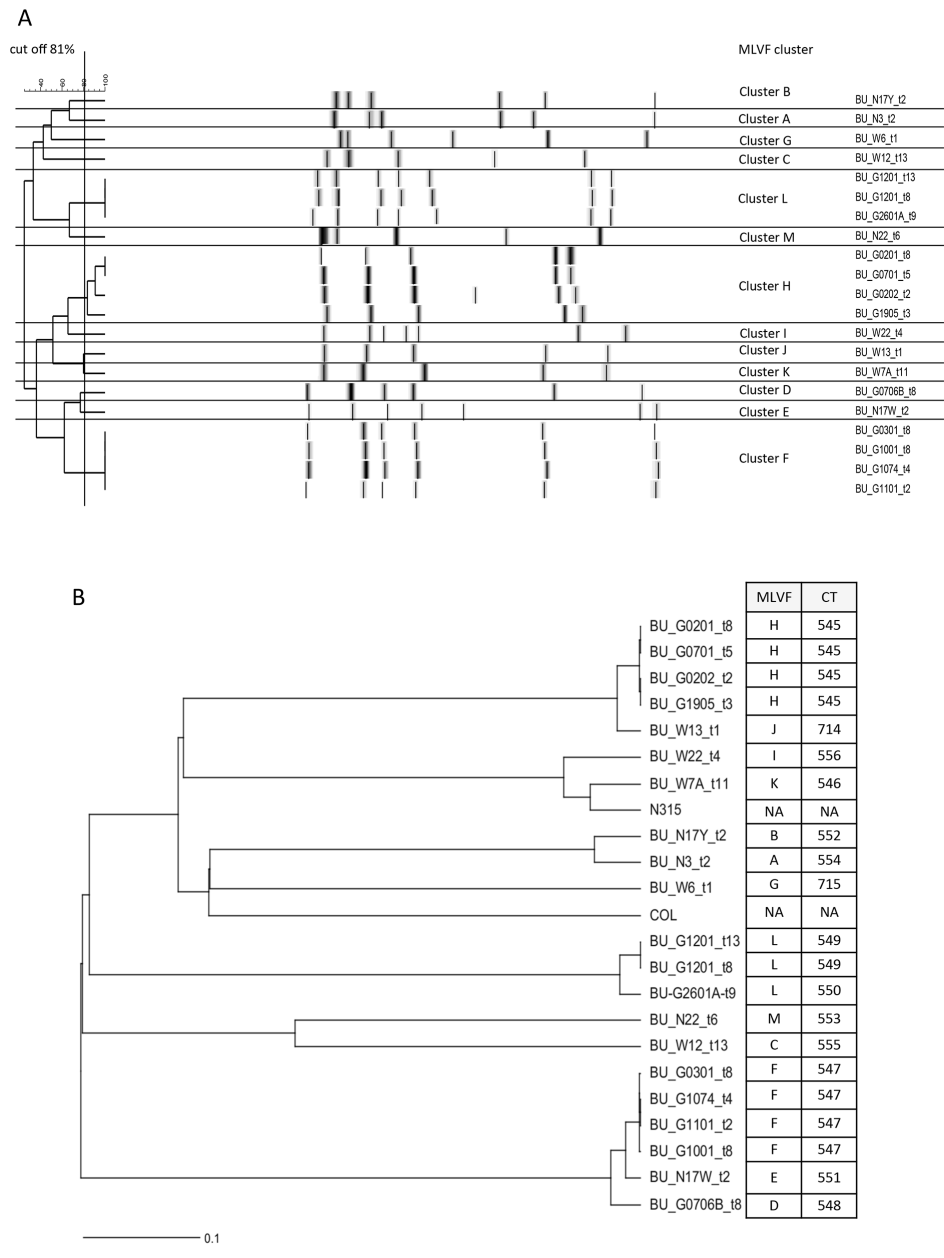
MLVF and SeqSphere^+^ dendrograms of 21 *S*. *aureus* isolates from BU patients. The dendrograms were generated using the UPGMA algorithm. (A), Dendrogram showing the previously identified MLVF clusters A-M. (B), SeqSphere^+^ dendrogram showing the 14 CTs identified in the present study. The reference strains COL (GenBank accession no. NC_002951) and N315 (GenBank accession no. BA000018.3) were included in the SeqSphere^+^ dendrogram. Isolates (BU_G0201_t8 and BU_G0202_t2 belonging to CT 545 and BU_G1074_t4 and BU_G1001_t8 belonging to CT 547) originating from patients 2 and 10 at two different time points are included in both transmission events. NA means ‘Not Applicable’.

In this study a transmission event is defined to have occurred if the wound of a patient, previously not containing a particular *S*. *aureus* genotype, becomes colonized over time by an *S*. *aureus* with a genotype that is identical with the genotype of an *S*. *aureus* isolate collected from the wound of another patient. Here we investigated whether transmission events had indeed occurred during wound care of patients treated in the Pakro Health Center, using the SeqSphere^+^ scheme which assigned each *S*. *aureus* isolate an allelic typing profile as previously described. The typing profile will, subsequently, be known as cluster type (CT). Hence a transmission event would have occurred if *S*. *aureus* isolates from different BU patients are grouped within the same CT. In our previous study, *S*. *aureus* isolates from BU out-patients, who visited the health center for wound care, were suspected to be involved in patient-to-patient transmission events [3]. These isolates were initially grouped by MLVF into clusters H and F (Fig 1A).

### Screening for antimicrobial resistance

*De novo* assembled genome sequences of *S*. *aureus* isolates were queried against specific previously identified sequence features, or compared to complete *S*. *aureus* reference genomes with associated annotated genes (S1 Table) using blastN in the WebACT comparison tool with default settings (http://www.webact.org/WebACT/prebuilt#). Further detailed analyses were performed with the Artemis Comparison Tool (ACT) software [23]. Specifically sequence data were queried for the presence of SCC*mec* elements and AMR genes. Similarity matches were filtered based on their length and percentage similarity scores, and only the filtered hits with at least 80% sequence similarity were then displayed by ACT and analyzed in detail. The AMR genes that were screened confer resistance to chloramphenicol, clindamycin, erythromycin, fusudic acid, kanamycin, lincosamide, methicillin, mupirocin, penicillin, rifampicin, streptogramin A and B, tetracycline, trimethoprim, tobramycin, and/or vancomycin. Antibiotic resistance profiles of the sequenced isolates were previously determined using vitek according to the EUCAST guidelines [3].

## Results

### Phylogeny of sequenced *S*. *aureus* isolates found in BU patients in Ghana based on a gene-by-gene comparison

From a total of 13 BU patients who visited the Pakro Healthcare Center for wound care 21 *S*. *aureus* isolates from the anterior nares (n = 4) and wounds (n = 17) were sequenced. These included six methicillin resistant MRSA and 15 methicillin susceptible *S*. *aureus* (MSSA) isolates. These isolates have been previously characterized by MLVF and *spa*-typing as shown in Table 2 [3].

**Table 2:** Genotypic and phenotypic characteristics of 21 *S*. *aureus* isolates from BU patients.

Patient No.	Sample ID	Start of treatment	End of wound care	Week of sampling	MLVF	CT	ST	*spa-*type	SCCmec	CXT	OXA	CIP	TET	CHL	TRI	STM	RIF
2	BU_G0201_t8	1-12-2012	Not yet	8	H	545	88	t786	IVa	+	*mecA*		*tetM*, *tetL*	*fexB*			
7	BU_G0701_t5	26-12-2012	25-6-2013	5	H	545	88	t786	IVa	+	*mecA*		*tetM*, *tetL*	*fexB*			
2	BU_G0202_t2	1-12-2012	Not yet	2	H	545	88	t786	IVa	+	*mecA*		*tetM*, *tetL*	*fexB*			
19	BU_G1905_t3	5-1-2013	22-7-2013	3	H	545	88	t786	IVa	+	*mecA*		*tetM*, *tetL*	*fexB*			
13	BU_W13_t1	12-12-2012	26-4-2013	1	J	714	88	t186	IVa	+	*mecA*		*tetM*, *tetL*	*fexB*			
22	BU_W22_t4	12-12-2012	Not yet	4	I	556	5	t2724					*tetK*		*drfG*		
7	BU_W7A_t11	26-12-2012	25-6-2013	11	K	546	5	t002	none	+	+	*gyrA* *					
17	BU_N17Y_t2	28-12-2012	N/A	2	B	552	15	t346							*drfG*		
3	BU_N3_t2	6-12-2012	N/A	2	A	554	15	t084					*tetM*				
6	BU_W6_t1	12-12-2012	26-4-2013	1	G	715	1	t7835					*tetK*	*fexB*			
12	BU_G1201_t13	19-12-2012	25-6-2013	13	L	549	121	t314					*tetK*	*catA*	*drfG*		
12	BU_G1201_t8	19-12-2012	25-6-2013	8	L	549	121	t314					*tetK*	*catA*	* drfG*		
26	BU_G2601A_t9	6-3-2013	21-6-2013	9	L	550	121	t314					*tetK*		*drfG*		*rpoB* *
22	BU_N22_t6	12-12-2012	N/A	6	M	553	3019	t939						*catA*			
12	BU_W12_t13	19-12-2012	25-6-2013	13	C	555	508	t12836									
3	BU_G0301_t8	6-12-2012	Not yet	8	F	547	152	t355					*tetK*	*catA*		*str*	
10	BU_G1074_t4	12-12-2012	26-4-2013	4	F	547	152	t355					*tetK*	*catA*		*str*	
11	BU_G1101_t2	19-12-2012	5-3-2013	2	F	547	152	t355					*tetK*				
10	BU_G1001_t8	12-12-2012	26-4-2013	8	F	547	152	t335					*tetK*	*catA*		*str*	
17	BU_N17W_t2	28-12-2012	N/A	2	E	551	152	t11375									
7	BU_G0706B_t8	26-12-2012	25-6-2013	8	D	548	152	t1096					*tetK*				

CXT—cefoxitin, OXA—oxacillin, CIP—ciprofloxacillin, TET—tetracycline, CHL–chloramphenicol, TRI–trimethoprim, STM–streptomycin and RIF—rifampicin.

The ‘*’ symbol indicates a mutation in a core genome gene that is involved in antibiotic resistance. All genes are involved in specific resistance phenotypes.

**Fig 2:**
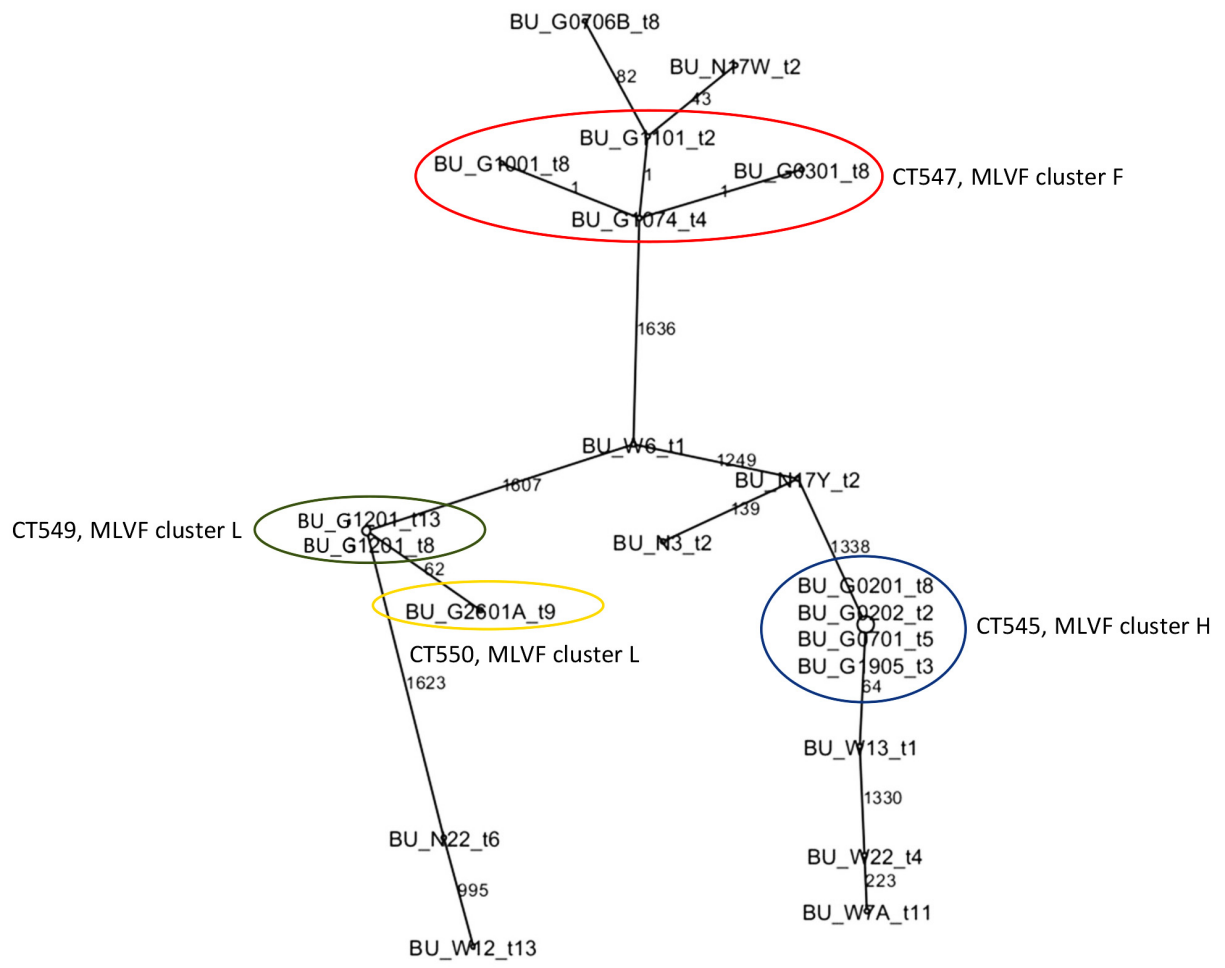
Minimum spanning tree showing the allelic difference between 21 *S*. *aureus* isolated from BU patients. The tree was generated using the SeqSphere^+^ software. The node size in the tree is proportional to the frequency of genotype occurrence. The allelic difference between *S*. *aureus* isolates is indicated as numbers between each node. Isolates with blue and red circles belonging to CTs 545 (MLVF cluster H) and 547 (MLVF cluster F) confirm transmission events. Isolates with green and yellow circles belonging to CTs 549 and 550 were initially grouped in MLVF cluster L and do not confirm transmission events.

A dendrogram was generated after SeqSphere^+^ analysis of the 21 sequenced isolates, which revealed 14 cluster types (CTs) (Figs 1B and 2) denoted as 545 (n = 4), 714 (n = 1), 556 (n = 1), 546 (n = 1), 552 (n = 1), 554 (n = 1), 715 (n = 1), 549 (n = 2), 550 (n = 1), 553 (n = 1), 555 (n = 1), 547 (n = 4), 551 (n = 1) and 548 (n = 1). This clustering by SeqSphere^+^ seemed to match well with the previous clustering of isolates by MLVF. To calculate the concordance between the SeqSphere^+^ and MLVF typing data, the Ridom epicompare software 1.0 (Ridom GmbH) was used with the Rands Adjusted co-efficient. This revealed a concordance of 0.924.

The 21 sequenced isolates were assigned to eight MLST types, namely ST1, ST5, ST15, ST88, ST121, ST152, ST508, and the new ST3019. The ST3019 is a single-locus variant of ST45 at the *yqiL* locus.

### Evidence of patient transmission events

A first transmission event was identified for four MRSA isolates belonging to ST88, which were previously grouped in the MLVF cluster H (Fig 1A) [3]. These isolates were classified by SeqSphere^+^ as CT 545 (Fig 1B). Within CT 545 the allelic profiles were identical (Fig 2). Of note, the four isolates were obtained from three different patients visiting the healthcare center over a period of seven months. The medical care for these patients involved antibiotic treatment and wound dressing changes (Tables 1 and 2). This particular MRSA was first identified in the wound of patient 2, who tested negative at the first sampling time point (t1). Patient 2 was the first to start treatment in this study and was found to carry this particular *S*. *aureus* genotype at several sampling time points during treatment (i.e. at t2, t3, t8 and t9). Patients 7 and 19 started treatment 25 and 35 days later, respectively. They both visited the health care center for wound care at time point t2. The wounds of patients 7 and 19 tested positive for *S*. *aureus* with the genotype of CT 545 at the sampling time points t5 (patient 7) and t3 (patient 19; Table 1), which is indicative of transmission events.

A second suspected transmission event was initially identified by MLVF typing (cluster F) and involved eight BU patients [3]. To investigate this possible transmission event in more detail four of the 25 isolates obtained from three patients were randomly selected and sequenced. These four MSSA ST152 isolates were assigned to CT 547 (Fig 1B). The allelic profiles within this cluster differed by one (Fig 2). Patients 10 and 11 tested positive for *S*. *aureus* with this particular genotype at the same sampling time point (t2). Patient 10 remained positive for *S*. *aureus* with the CT 547 until sampling time point t8, and patient 11 until time point t5 (Table 1). A third patient (patient 3) was found to be positive for *S*. *aureus* with the CT 547 at sampling time point t8 (Table 1). Patients 5, 6, 7, 18 and 24 became positive for *S*. *aureus* with this genotype at later time points than patients 10 and 11. The patients 5, 6, 7, 18 and 24 paid at least one visit at the health center for wound care that overlapped with visits by three other patients, which were found to be positive for the *S*. *aureus* genotype with the CT 547 (Table 1).

It is noteworthy to mention that in each of the transmission events, the gene allele variation between isolates was not higher than one. This implies that the isolates were nearly identical with respect to their core genome.

### Antibiotic resistance genes

The assembled genomes of the 21 *S*. *aureus* isolates were used in blast comparisons to detect the presence of AMR genes, and the results are shown in Table 2. Among the investigated isolates none was found to carry genes involved in resistance to erythromycin, fusidic acid, kanamycin, mupirocin, or vancomycin. Antibiotic resistance of the sequenced isolates was previously most often found against penicillin, chloramphenicol, tetracycline and trimethoprim [3]. Consistent with their penicillin resistance, all sequenced isolates carried various types of *blaZ* operons, which were located on chromosomally integrated transposons or plasmids. Specifically, the *bla*Z gene was found in 16 isolates that belonged to ST1, ST5, ST15, ST88, ST152 and ST3019, while the *bla*Z-B variant was found in five isolates representing ST5, ST508 and ST121. Fourteen sequenced *S*. *aureus* isolates were chloramphenicol resistant of which six (ST121, ST3019 and ST152) carried various plasmids with a *catA* gene. Six other isolates (ST88 and ST1) carried a novel allele of *fexB* that was not previously reported in *S*. *aureus*. In the case of one isolate, the phenotypic resistance for chloramphenicol could not be confirmed at the genomic level, which was potentially due to the loss of the resistance gene. Resistance to rifampicin was identified in one isolate belonging to ST121 where the *rpoB* gene was found to encode an amino acid substitution that changed Asp471 into Gly. Resistance to tetracycline was identified in 16 isolates, which was confirmed by the identification of resistance genes, such as *tetK*, *tetL* and *tetM*. The *tetK* gene was located on plasmid pT181, which was found in 10 isolates representing different STs. Five isolates of ST88 contained the *tetL* and *tetM* genes located on identical mobile genetic elements integrated into their genomes, while one isolate of ST15 contained a transposon with *tetM*. The presence of a plasmid or transposon carrying the *drfG* gene responsible for trimethoprim resistance was detected in five isolates that belonged to ST5, ST15 and ST121. Resistance to streptomycin was limited to three isolates of ST152 where the *str* gene was present. Of the six methicillin resistant isolates, five belonging to ST88 contained the *mec*A gene, whereas one ST5 isolate contained neither *mecA* nor *mecC*. The latter isolate was termed borderline oxacillin resistant *S*. *aureus* (BORSA). Intriguingly, the BORSA isolate contained no mutations in the genes for the penicillin-binding proteins PBP1, PBP2, and PBP3 or the YjbH protein, which were previously proposed to be involved in BORSA phenotypes [24]. However, sequence comparisons revealed that the PBP2 protein of the BORSA isolate contains a Tyr residue at position 197, while the PBP2 protein of *S*. *aureus* N315 contains a Cys residue at this position. Furthermore, the BORSA isolate showed resistance to fluoroquinolones, which may be due to a specific mutation in the gyrase A gene (Ser84Leu).

## Discussion

In the present study, we have investigated *S*. *aureus* transmission events in BU patients during wound care by implementing a WGS-based gene-by-gene typing approach using SeqSphere^+^. The SeqSphere^+^ scheme grouped the 21 sequenced *S*. *aureus* isolates into eight different STs. Sequenced *S*. *aureus* that belonged to ST88 isolates shared identical characteristics (*spa*-types t186/t786, SCC*mec* type IVa, PVL-negative) with isolates collected from out-patients in Egypt and Angola, indicating a larger geographic distribution on the African continent [25,26]. The new ST3019 (*spa*-type t939) identified in this study belongs to the same clonal complex (CC45) as ST45 and ST508. Compared to ST45 a single locus variation was observed at the *yqiL* locus for ST3019 and in the *aroE* locus for ST508.

Using SeqSphere^+^, we identified two major clusters of *S*. *aureus* isolates from different BU patients, which may reflect transmission events that occurred during overlapping visits to the Pakro Healthcare Center where these patients received wound care. None of these patients carried *S*. *aureus* with the CTs 545 or 547 on their first visit to the Pakro Healthcare Center, strongly suggesting that they acquired the respective *S*. *aureus* types upon wound care. Interestingly, the majority of *S*. *aureus* isolates from BU patients belong to lineages characterized by *spa*-types t786 and t355 that have been already reported in health care settings in Ghana [11]. This suggests the nosocomial acquisition of these *S*. *aureus* types by patient-to-patient transmission between BU patients and healthcare workers that may have occurred due to inadequate hygiene. Indeed, it has been reported in a recent study that 8 of 11 MRSA transmission events among patients in intensive care settings were potentially due to poor hand hygiene [16]. This could probably be avoided by wearing gloves and protective gowns, and strict implementation of hand hygiene [27–29]. Basic preventive measures, such as adherence to aseptic techniques may further reduce the risk of infection thereby improving wound care of patients, provided that gloves, gowns, adequate dressing materials, running water and hand rub alcohol are made available. With a steady supply and stock of equipment and disposables, routine screening of patients and healthcare workers for *S*. *aureus* may be less critical.

Genotypic data of the isolates sequenced confirmed the results of the antimicrobial resistance profiles described previously [3]. Interestingly, the chloramphenicol resistance of some isolates was conveyed by the *fexB* gene (Table 2), which was thus far not encountered in *S*. *aureus*. On the other hand, *fexB* was previously reported in *Enterococcus faecium* EFM-1 and *Enterococcus hirae* EH-1 isolates from pigs [30]. As Enterococci were previously identified in the wounds of BU patients, it is conceivable that the MRSA isolates acquired the *fexB* gene by horizontal gene transfer from such Enterococci [3]. Furthermore, a BORSA phenotype was identified in an isolate belonging to ST5. Such a BORSA phenotype was previously reported for *S*. *aureus* isolates with ST1, ST8 and ST15 that were implicated in wound infections in Scotland [24]. The presence of specific mutations in the genes coding for four proteins, namely PBP1, PBP2, PBP3 and YjbH, was proposed to be involved in the BORSA phenotype. However, in the genome sequence of the presently investigated BORSA isolate from a BU patient, none of these mutations was found. After genomic comparison of the BORSA isolate with the N315 reference genome, the only difference was observed for PBP2, where at position 197 a cysteine residue was replaced by a tyrosine residue. However, this PBP2 amino acid substitution is encoded by the majority of *S*. *aureus* genomes available in the NCBI database and, therefore, it may not explain the BORSA phenotype observed. Further comparative genome analyses revealed about 300 additional non-synonymous SNPs, which could contribute to the observed BORSA phenotype.

In summary, WGS of *S*. *aureus* isolates from BU patients and the subsequent analysis of sequencing data using the SeqSphere^+^ scheme revealed likely patient-to-patient transmission events in a healthcare setting in Ghana. This indicates a need for the implementation of improved hygiene protocols in healthcare settings where BU patients receive wound care. Apart from the detection of transmission events, WGS has the advantage that it also provides information on antimicrobial resistance. Related to the antimicrobial resistance pheno- and genotypes identified in *S*. *aureus* isolates from BU patients, it is important to bear in mind that antimicrobial pressure has the potential to aggravate resistance, with an inherent risk for transmission of resistant organisms. Therefore, even in low-resource settings, antimicrobial stewardship programs are likely to have added value, with more restrictive antimicrobial use than currently practiced [2].

## Supporting Information

S1 Table Information on reference genomes from which the antibiotic resistance genes were queried. (DOCX)
