# Supplementary material for: Molecular Characterization of Staphylococcus aureus Isolates Transmitted between Patients with Buruli Ulcer
Source: PLoS Negl Trop Dis. 2015 Sep 11;9(9):e0004049. doi: 10.1371/journal.pntd.0004049 (PMC4567303; doi:10.1371/journal.pntd.0004049)
Supplement: S1 Table — (DOCX) [file pntd.0004049.s001.docx]

S1 Table. Information on reference genomes from which the antibiotic resistance genes were queried

| **Gene** | **Accession number** | **Reference genome (location)** | **Reference** |
| --- | --- | --- | --- |
| *mecA* | GenBank: CP000046.1 | *S. aureus* COL (39643 – 41649) | Gill et al. 2005 |
| *tetK* | GenBank: CP000045.2 | *S. aureus* COL; plasmid pT181 (1138 – 2517) | Gill et al. 2005 |
| *tetM* | GenBank: AP009324.1 | *S. aureus* Mu3 (439868 – 441787) | Hiramatsu et al. 1997 |
| *tetL* | GenBank: FN377602.2 | *S. aureus* plasmid pkkS825 (5846 - 7225) | Kadlec et al. 2009 |
| *catA* | GenBank: X02529.1 | *S. aureus* plasmid pC221 (2267 – 2914) | Projan et al. 1985 |
| *fexB* | GenBank: CP002208.2 | Enterococcus faecalis plasmid pEP-01 (10637 – 12046) | Liu et al. 2013 |
| *drfG* | GenBank: FN433596.1 | *S. aureus* TW20 (502263 – 502760) | Holden et al. 2010 |
| *str* | GenBank: AM990994.1 | *S. aureus* SO385; plasmid pSO385-2 (155 – 1033) | Schijffelen et al. 2010 |
| *blaZ* | GenBank: FN433596.1 | *S. aureus* TW20 (2879866 – 2880711) | Holden et al. 2010 |
| *blaZ-B* | GenBank: AF086644.1 | *S. aureus* 2260 (89 – 820) | Voladri et al. 1998 |
| *rpoB* | GenBank:CP000253.1 | *S. aureus* NCTC8325 (522301-525711) | Aubry-Damon et al. 1998 |
